# Human-like collagen protein-coated magnetic nanoparticles with high magnetic hyperthermia performance and improved biocompatibility

**DOI:** 10.1186/s11671-015-0752-3

**Published:** 2015-01-31

**Authors:** Xiaoli Liu, Huan Zhang, Le Chang, Baozhi Yu, Qiuying Liu, Jianpeng Wu, Yuqing Miao, Pei Ma, Daidi Fan, Haiming Fan

**Affiliations:** Shaanxi Key Laboratory of Degradable Biomedical Materials, School of Chemical Engineering, Northwest University, Taibai North Road 229, Xi’an, Shaanxi 710069 China; Department of Materials Science and Engineering, Faculty of Engineering, National University of Singapore, 7 Engineering Drive 1, Singapore, 117574 Singapore; School of Chemical Engineering, Northwest University, Taibai North Road 229, Xi’an, Shaanxi 710069 China; Institute of Photonics & Photon-Technology, Northwest University, Taibai North Road 229, Xi’an, Shaanxi 710069 China

**Keywords:** Human-like collagen, Magnetic nanoparticles, Magnetic hyperthermia, Biocompatibility

## Abstract

Human-like collagen (HLC)-coated monodispersed superparamagnetic Fe_3_O_4_ nanoparticles have been successfully prepared to investigate its effect on heat induction property and cell toxicity. After coating of HLC, the sample shows a faster rate of temperature increase under an alternating magnetic field although it has a reduced saturation magnetization. This is most probably a result of the effective heat conduction and good colloid stability due to the high charge of HLC on the surface. In addition, compared with Fe_3_O_4_ nanoparticles before coating with HLC, HLC-coated Fe_3_O_4_ nanoparticles do not induce notable cytotoxic effect at higher concentration which indicates that HLC-coated Fe_3_O_4_ nanoparticles has improved biocompatibility. Our results clearly show that Fe_3_O_4_ nanoparticles after coating with HLC not only possess effective heat induction for cancer treatment but also have improved biocompatibility for biomedicine applications.

## Background

Recently, magnetic nanoparticles (NPs) have been applied in many biomedicine fields because of their appealing magnetic properties [1-4]. In particular, magnetic NPs could be used as magnetic hyperthermia agents for the treatment of cancer [5,6]. Until now, superparamagnetic Fe_3_O_4_ NPs, the only clinically approved metal NPs, are widely used in these bio-related investigations because of their superparamagnetism, large specific surface area, and enhanced reactivity [7]. Although superparamagnetic NPs offer rapid growth and therapeutic benefits, at the same time, there are risks and concerns related with their exposure to cells. Therefore, there is a considerable need to address biocompatibility and biosafety concerns associated with their usage in a variety of applications.

Several studies have been reported that the mechanism of toxicity induced from NPs is mainly because of the generation of reactive oxygen species (ROS), which could indirectly damage DNA, proteins, and lipids and results in cell death [8-10]. To date, significant improvements to the cell toxicity of superparamagnetic NPs have been made. Various biocompatible surfactants or polymers have been applied for surface modification of superparamagnetic NPs to reduce its toxicity. For example, albumin-derived superparamagnetic NPs did not result in cell death compared with uncoated superparamagnetic NPs [11,12]. Uncoated superparamagnetic NPs induced greater toxicity compared to that of NPs after coating with biocompatible polyvinyl alcohol (PVA) [13]. Citrate-coated superparamagnetic NPs have been shown to lead to cellular oxidative stress in rat macrophages without causing any toxicity effects [14]. However, certain sizes of superparamagnetic NPs possess low performance of magnetic hyperthermia after being optimized by surface coating for its biocompatible and soluble. This could be due to the low saturation magnetization and reduced contribution of Brown relaxation on heat after surface modification [5]. Moreover, nanoparticles are not well dispersed after being coated with the polymer, which also influences the performance of magnetic hyperthermia. It is critical to optimize magnetic NPs for high heat transfer efficiency and at the same time possess good biocompatibility and colloidal stability in aqueous solution. Hence, it is imperative to design superparamagnetic NPs with specifically tailored surface to meet the demands of the rapidly proliferating field of magnetic hyperthermia application.

Recently, much effort has been expended to design biomaterials which can offer biocompatibility, especially the engineered human-like collagen (HLC). HLC is a special protein and is expressed by recombinant *Escherichia coli* with a modified cDNA fragment transcribed from the mRNA coding for human collagen [15,16]. Different from animal-derived collagen, HLC has excellent biocompatibility and can easily dissolve in aqueous solutions [17,18]. However, to the best of the authors’ knowledge, there are few cases reported about the HLC modified superparamagnetic NPs to be used as magnetic hyperthermia agents for cancer treatment. In the present study, highly monodispersed Fe_3_O_4_ NPs are employed to study the effect of HLC-coated Fe_3_O_4_ NPs on both the efficiency of magnetic hyperthermia and cell toxicity. This paper represents one of the first attempts at investigating the effect of HLC-coated superparamagnetic NPs on the magnetic hyperthermia performance and its biocompatibility.

## Methods

### Materials

Hexane (J.T. Baker, 99.0%; Avantor Performance Materials, Inc., Center Valley, PA, USA) and absolute ethanol were used as received. Ethyl acetate (99.5%) was purchased from Fluka (St. Louis, MO, USA). Iron (III) acetylacetonate (Fe(acac)_3_; 97.9%), benzyl ether (99%), oleic acid (90%), acetonitrile (≥99.0%), and sodium periodate (≥99.8%) were purchased from Aldrich Chemical Co. (St. Louis, MO, USA ).

### Preparation of highly monodispersed Fe_3_O_4_ NPs

As described previously [5,19], high-quality Fe_3_O_4_ NPs were synthesized by high-temperature thermal decomposition method. Under a flow of nitrogen, Fe(acac)_3_ (6 mmol), oleic acid (20 mmol), and benzyl ether (50 mL) were mixed by magnetic stirring. The mixture was first heated to 165°C for 30 min and then heated to 280°C for refluxing under a nitrogen atmosphere for another 30 min. Finally, the mixture was allowed to cool down to room temperature naturally. Ethanol (40 mL) was then added to the mixture under ambient conditions. The product was separated by centrifugation and re-dispersed into hexane.

### Transfer of Fe_3_O_4_ NPs into water

The transfer of Fe_3_O_4_ NPs from hexane to water was through a simple method, which is by oxidation of oleic acid [20,21]. Firstly, the mixture of ethyl acetate and acetonitrile at 1:1 volume ratio was added into the hexane containing as-synthesized Fe_3_O_4_ NPs (10 mg). Sodium periodate aqueous solution (40 mg per 1.5 mL) as oxidative agent was then added under vortex mixture. After 2 h, the upper hexane layer was discarded and the aqueous solution at the bottom was magnetically separated. After repeated washing with distilled water (three times), the obtained Fe_3_O_4_ NPs were re-dispersed in water.

### Coating of HLC on the surface of hydrophilic Fe_3_O_4_ NPs

Coating of HLC on the surface of hydrophilic Fe_3_O_4_ NPs was performed by using standard (1-ethyl-3-[3-dimethylaminopropyl]carbodiimidehydrochloride)/N-Hydroxysuccinimide (EDC/NHS).

### Characterization

The phase of as-synthesized Fe_3_O_4_ NPs was characterized by X-ray powder diffraction on a Bruker D8 Advanced Diffractometer System (Bruker AXS, Inc., Madison, WI, USA) equipped with Cu/Kα radiation in the 2*θ* range from 20° to 80° (*λ* = 1.5418 Å). The size and morphology of samples were characterized using a JEOL 100CX transmission electron microscope (TEM; JEOL Ltd., Akishima-shi, Japan). The mean particle size was obtained from TEM images by counting more than 100 particles. The structure of the particles was characterized using a high-resolution TEM (HRTEM) and selected area electron diffraction (SAED) on a JEOL100CX TEM. Dynamic light scattering (DLS) measurements were performed in a Malvern Zetasizer Nano-ZS device (Malvern, WR, UK) to determine the hydrodynamic size of Fe_3_O_4_ NPs before and after coating HLC in a colloidal suspension. The zeta-potential of the suspensions was measured at 25°C. UV-vis absorption spectra were taken using a Shimadzu UV-1601 UV-visible spectrophotometer (Shimadzu, Kyoto, Japan). Magnetic properties of the samples were characterized by a LakeShore Model 7407 vibrating sample magnetometer (VSM; Lake Shore Cryotonics Inc., Wersterville, OH, USA).

### Magnetic hyperthermia

Fe_3_O_4_ NPs before and after coating with HLC were dispersed in water. Thermally insulated plastic bottles containing 2 mL samples were placed within a water-cooled copper coil driven by an Inductelec A.C. generator (SPG-10AB-II; Shenzhen Magtech Company Limited, Shenzen, China). The applied frequency was 366 kHz, and the heating behavior of the samples was studied at field strength of 500 Oe. A Luxtron MD600 fiber optic thermometry unit (Luxtron Corporation, Santa Clara, CA, USA) connected to a computer was used to measure the sample’s temperature. The specific absorption rate (SAR) of the samples was calculated from the following equation [22]:

1 $$ \mathrm{S}\mathrm{A}\mathrm{R}=C\frac{\varDelta T}{\varDelta t}\frac{1}{m_{\mathrm{Fe}}}. $$

where *C* is the specific heat of the medium (*C*_water_ = 4.18Jg^−1^°C^−1^), Δ*T*/Δ*t* is the maximum slope of the time-dependent temperature curve, and *m*_Fe_ is the weight fraction of the magnetic element in the sample.

### Cytotoxicity assay

NIH3T3 cells were cultured in Dulbecco's modified Eagle medium (DMEM) supplemented with 10% fetal calf serum in 5% CO_2_ atmosphere at 37°C. Cells were seeded into a 96-well plate at a concentration of 8,500 cells/well. After 24 h, 20 μL magnetic suspensions with various Fe concentrations (25 to 250 μg/mL) were added to each well for co-incubation for 24 h. And then, CCK-8 (10 μL) was added to each well and the samples in the 96-well plate were further incubated for a further 4 h before the absorbance readings, which were conducted at 450 nm using FluoStar Optima microplate reader (LUOstar OPTIMA, BMG Labtech GmbH, Germany).

## Results and discussion

**Figure 1:**
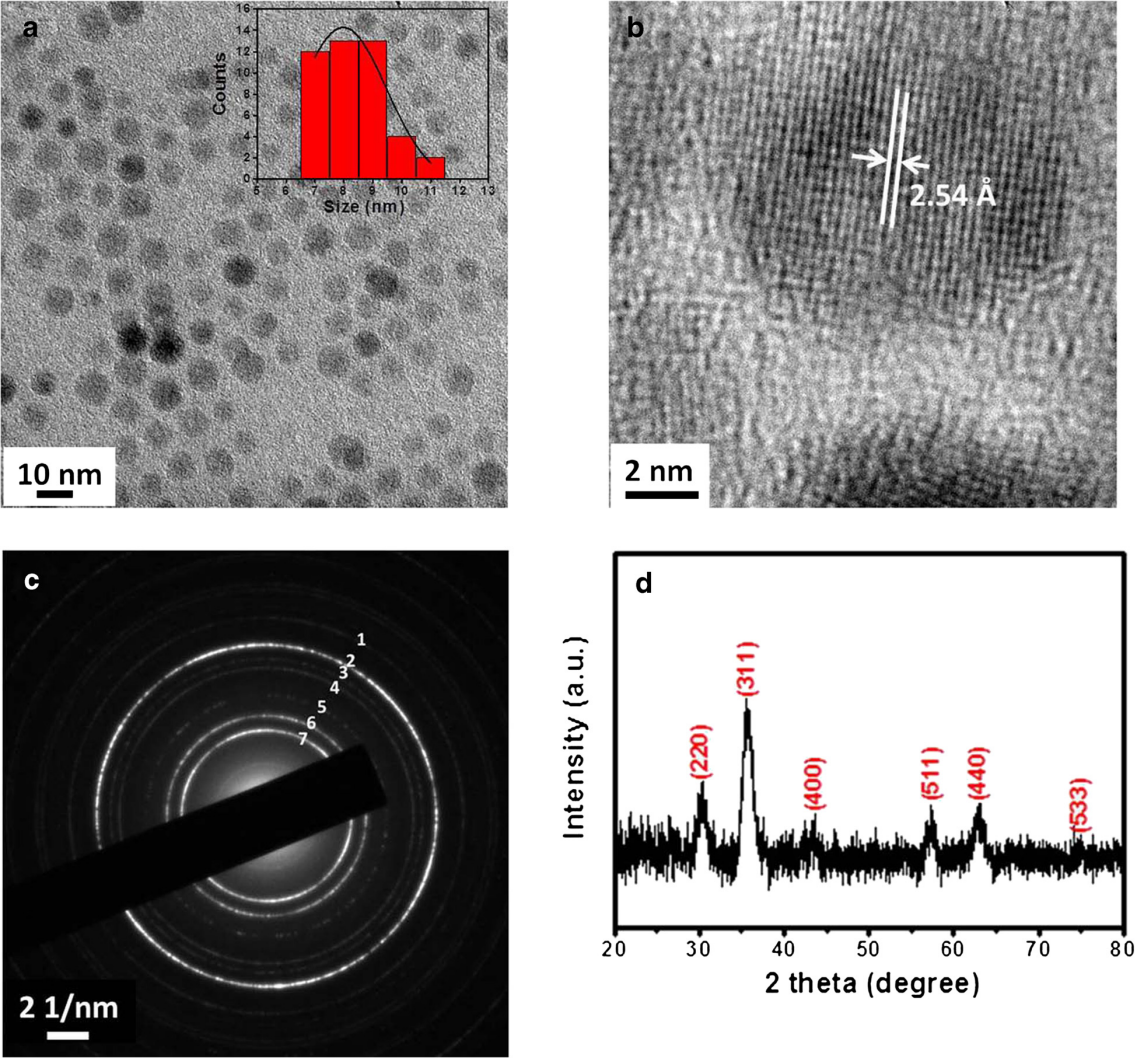
**Structural characterization of Fe**_**3**_ **O**_**4**_ **NPs. (a)** TEM image of the as-synthesized Fe_3_O_4_ NPs, insert shows the size distribution histogram. **(b)** High-resolution TEM image of a single Fe_3_O_4_ NPs. **(c)** Selected area electron diffraction (SAED) pattern acquired from Fe_3_O_4_ NPs assembly. **(d)** XRD pattern.

Highly monodispersed superparamagnetic Fe_3_O_4_ NPs were synthesized by well-established solution-phase high-temperature thermal decomposition of iron acetylacetonate. TEM images (Figure 1a) reveal that as-synthesized superparamagnetic Fe_3_O_4_ NPs are almost spherical with an average diameter of 8.4 nm. The particle size is fairly uniform with a narrow distribution (Figure 1a). The structural information from a single Fe_3_O_4_ NP is obtained using HRTEM. The lattice fringes in the HRTEM image (Figure 1b) correspond to a group of atomic planes within the particle, which is indicative of the high crystallinity of these particles. The distance between two adjacent planes is measured to be 2.54 Å, corresponding to (311) planes in the inverse spinel-structured Fe_3_O_4_. An assembly of Fe_3_O_4_ NPs is characterized by both electron and X-ray diffraction. Figure 1c is a SAED pattern acquired from as-synthesized Fe_3_O_4_ NPs assembly. Table 1 displays the measured lattice spacing based on the rings in the diffraction pattern and compares them to the lattice spacing for bulk Fe_3_O_4_ along with their respective hkl indexes from the PDF database. Figure 1d shows the powder XRD pattern of as-synthesized Fe_3_O_4_ NPs. It can be seen that the position and relative intensity of all diffraction rings/peaks match well with standard Fe_3_O_4_ powder diffraction data (JCPDS card no. 19-0629), which further indicates that Fe_3_O_4_ NPs synthesized by using this method is highly crystalline and can be used as a model for the further investigation. The distinct broadening of the diffraction peaks implies that the size of the as-synthesized Fe_3_O_4_ NPs is very small. By applying the Scherrer equation to the most intense peak of the XRD pattern, the mean crystallite size is estimated to be about 8 nm, which is consistent with that determined by statistical analysis of the TEM images.

**Table 1:** **Measured lattice spacing**

	**Ring**						
	**1**	**2**	**3**	**4**	**5**	**6**	**7**
*d*	1.33	1.51	1.64	1.77	2.13	2.57	3.02
Fe_3_O_4_	1.33	1.48	1.62	1.71	2.1	2.53	2.97
*hkl*	620	440	511	422	400	311	220

*d* (Å), based on the rings in Figure 1c and standard atomic spacing for Fe_3_O_4_ along with their respective *hkl* indexes from the PDF database.

**Figure 2:**
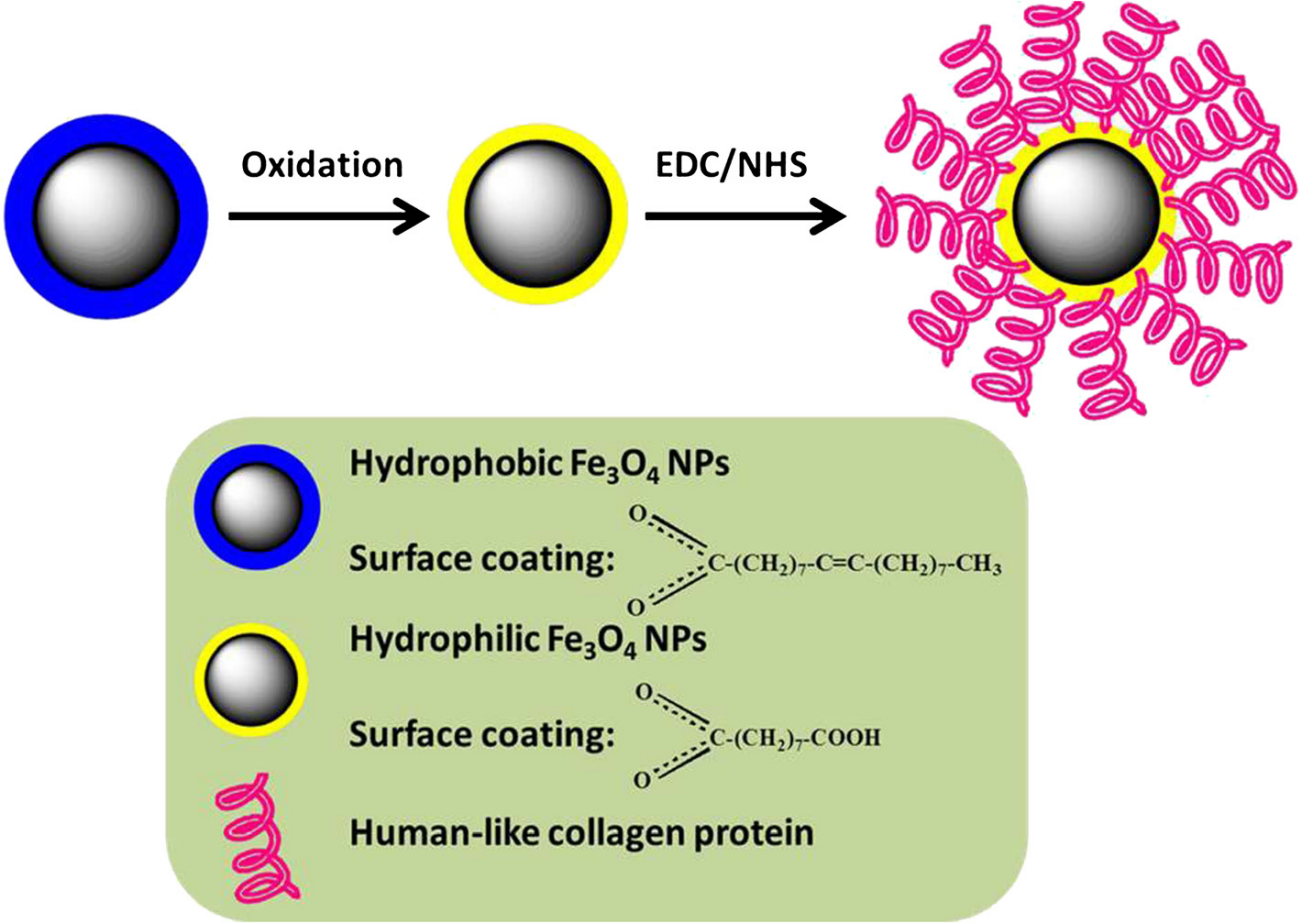
**Schematic diagram.** The strategy of coating HLC on the surface of Fe_3_O_4_ NPs.

**Figure 3:**
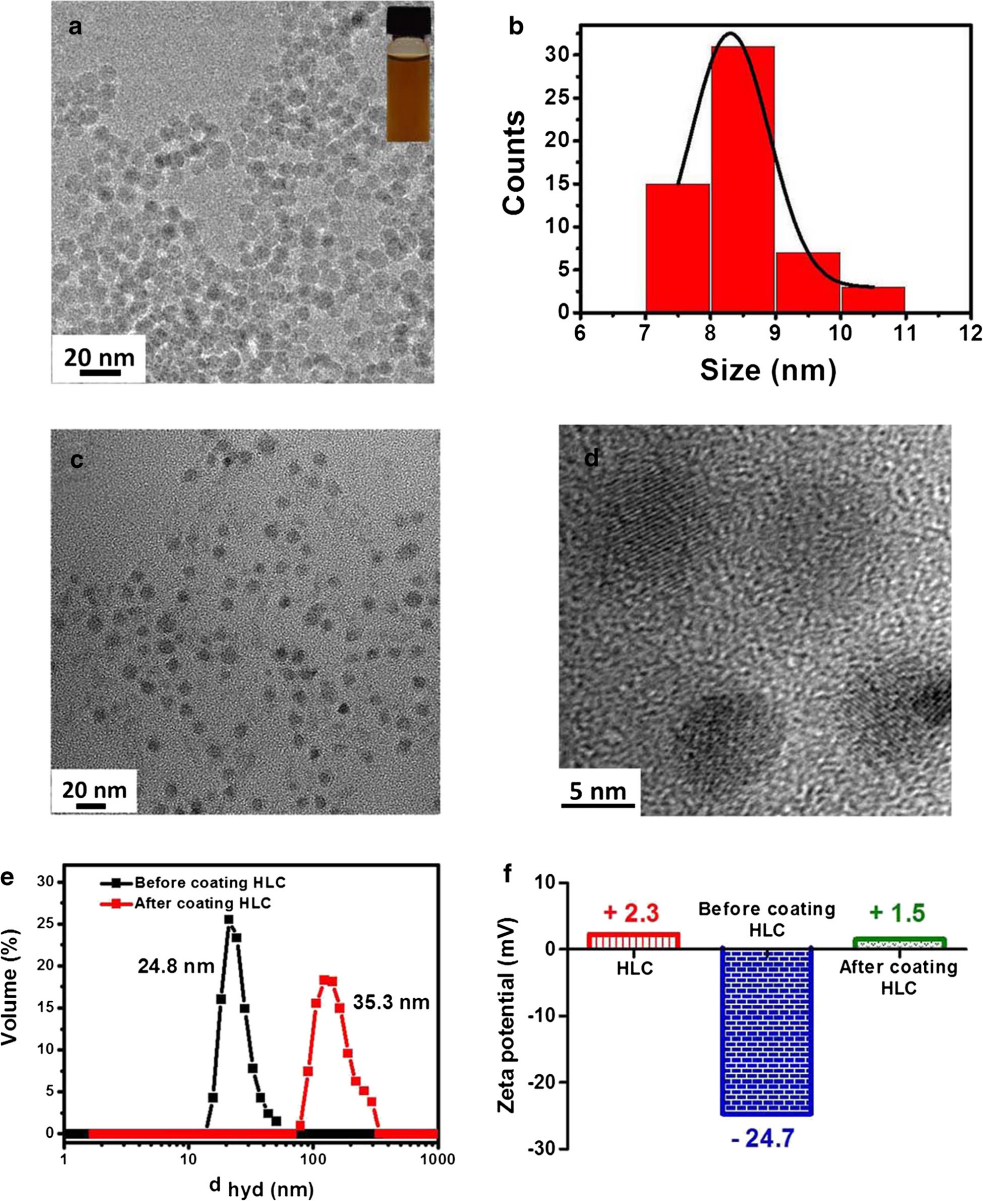
**TEM, DLS, and zeta-potential. (a)** TEM image of hydrophilic Fe_3_O_4_ NPs, insert shows digital photograph of hydrophilic Fe_3_O_4_ NPs water dispersions. **(b)** Size distribution histogram. **(c)** TEM image of Fe_3_O_4_ NPs after coating HLC. **(d)** High-resolution TEM image of Fe_3_O_4_ NPs after coating HLC. **(e)** Hydrodynamic diameter of Fe_3_O_4_ NPs before and after coating HLC. **(f)** The zeta-potential of Fe_3_O_4_ NPs before and after coating HLC.

**Figure 4:**
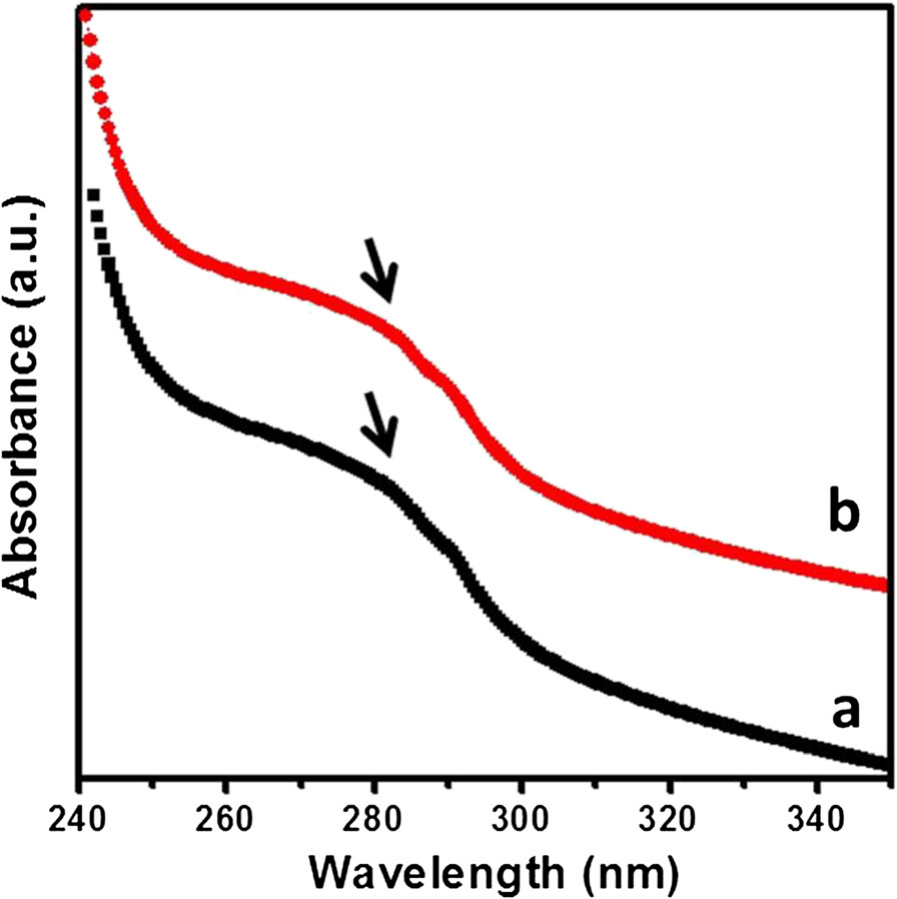
**UV-vis spectra.** UV-vis spectra of **(a)** HLC and **(b)** HLC-coated Fe_3_O_4_ NPs.

Figure 2 shows the schematic diagram illustrating the method of coating HLC on the surface of as-synthesized Fe_3_O_4_ NPs. As-synthesized uniform Fe_3_O_4_ NPs cannot be dispersed in water solution because of oleic acid (OA) coated on its surface, which largely restricts their subsequent biomedicine applications. It is necessary to first disperse these hydrophobic Fe_3_O_4_ NPs in aqueous media before they can be used for biomedical applications. To preserve the morphology of the NPs, avoid low exchange efficiency and also avoid using expensive customized copolymers and surfactants; here, the Fe_3_O_4_ NPs were dispersed in aqueous solution by oxidation and decomposition of OA which was chem-absorbed on the surface of NPs. By using this method, the Fe_3_O_4_ NPs can be made to be hydrophilic and consequently dispersed in water. More importantly, in this way, it will produce the azelaic and pelargonic acids with carboxyl group [23], which may functionalize nanoparticles with the following HLC by using standard (1-ethyl-3-[3-dimethylaminopropyl]carbodiimidehydrochloride)/N-Hydroxysuccinimide (EDC/NHS). Figure 3a shows the TEM image of Fe_3_O_4_ NPs dispersion in water solution. It can be seen that the size and shape do not change after dispersing into water. As shown in Figure 3b, the average size is about 8.2 nm and also with a narrow size distribution. The photograph inserted in Figure 3a shows that the Fe_3_O_4_ NPs dispersion is quite clear and no obvious aggregation. HLC was then coated on the surface of hydrophilic Fe_3_O_4_ NPs by using standard EDC/NHS method. TEM and HRTEM image (Figure 3c,d) show that Fe_3_O_4_ NPs are still monodispersed and with high crystalline after coating with HLC. After coating with HLC, the surface property of Fe_3_O_4_ NPs is changed. DLS measurements were carried out to evaluate the hydrodynamic diameter of the Fe_3_O_4_ NPs dispersion. As shown in Figure 3e, Fe_3_O_4_ NPs before coating with HLC has a hydrodynamic size of 24.8 nm, which is considerably larger than that observed using TEM. Such differences in the mean diameters have also been observed for other nanomaterials [3,24]. After coating with HLC, the hydrodynamic size becomes 35.5 nm, which is obviously larger than that of the uncoated hydrophilic Fe_3_O_4_ NPs. Moreover, the hydrodynamic size of HLC-coated Fe_3_O_4_ NPs determined by DLS does not change significantly for 1 month, further proving the excellent stability of these HLC-coated Fe_3_O_4_ NPs for biomedicine applications. The surface charge properties of Fe_3_O_4_ NPs before and after coating were studied by measuring the zeta potentials as a function of pH values. Figure 3f shows the surface charges (zeta-potential) of the corresponding Fe_3_O_4_ NPs at neutral pH value (pH = 7). HLC itself has a zeta-potential of +2.3 mV. Before coating HLC, it shows −24.7 mV. After coating HLC, it becomes +1.5 mV, which results from the attachment of positive HLC on the surface. These observations clearly indicate the presence of HLC on the surface of Fe_3_O_4_ NPs. The HLC-coated Fe_3_O_4_ NPs were further characterized by UV-vis absorbance to verify the formation of the HLC coating. Figure 4a,b shows the UV-vis absorption spectra of HLC and HLC-coated Fe_3_O_4_ NPs, respectively. The absorption band is at 280 nm, which is attributed to the absorbance of tyrosine [25]. This peak is present in HLC-coated Fe_3_O_4_ NPs, which implies that HLC is capped on the surface of Fe_3_O_4_ NPs.

**Figure 5:**
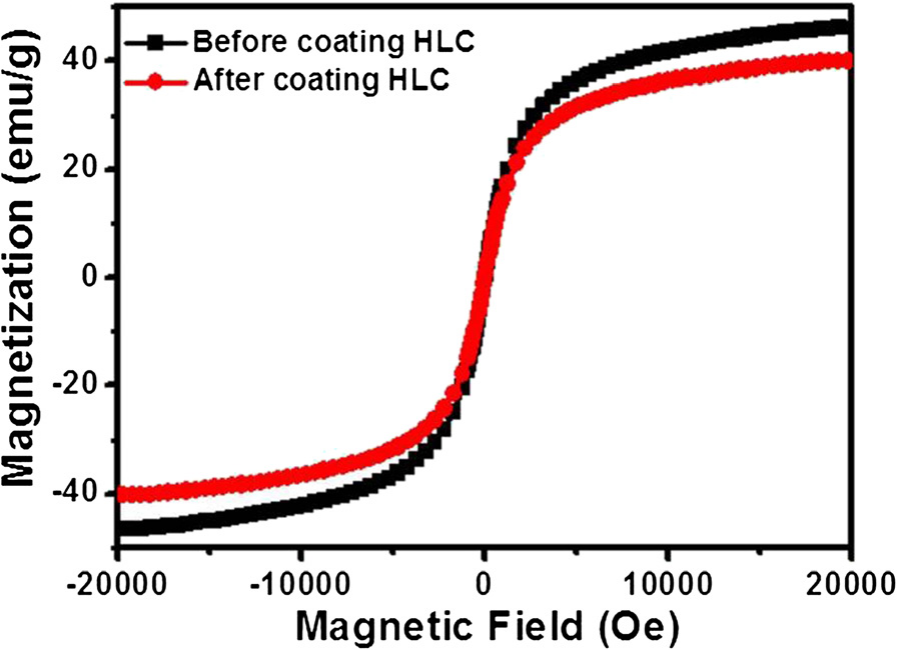
**VSM characterization.** The hysteresis loop of Fe_3_O_4_ NPs before (black cubic) and after (red circle) at room temperature.

For clinical application of HLC-coated Fe_3_O_4_ NPs as a high performance magnetic hyperthermia agent, it is critical that HLC-coated Fe_3_O_4_ NPs should maintain their magnetic properties after coating with HLC. The magnetic properties of HLC-coated Fe_3_O_4_ NPs were characterized by using a VSM. The Fe_3_O_4_ NPs exhibit superparamagnetic properties at room temperature before and after coating with HLC, with no coercivity and remanence. As shown in Figure 5, the saturation magnetizations (*M*_*s*_) before and after coating with HLC are 46 and 40 emu/g, respectively. The reduced *M*_*s*_ after coating with HLC is mainly attributed to the decreased effective weight fraction of the magnetic core. However, since the variation of *M*_*s*_ was within 15%, this suggests that coating with HLC does not significantly change the magnetic properties.

**Figure 6:**
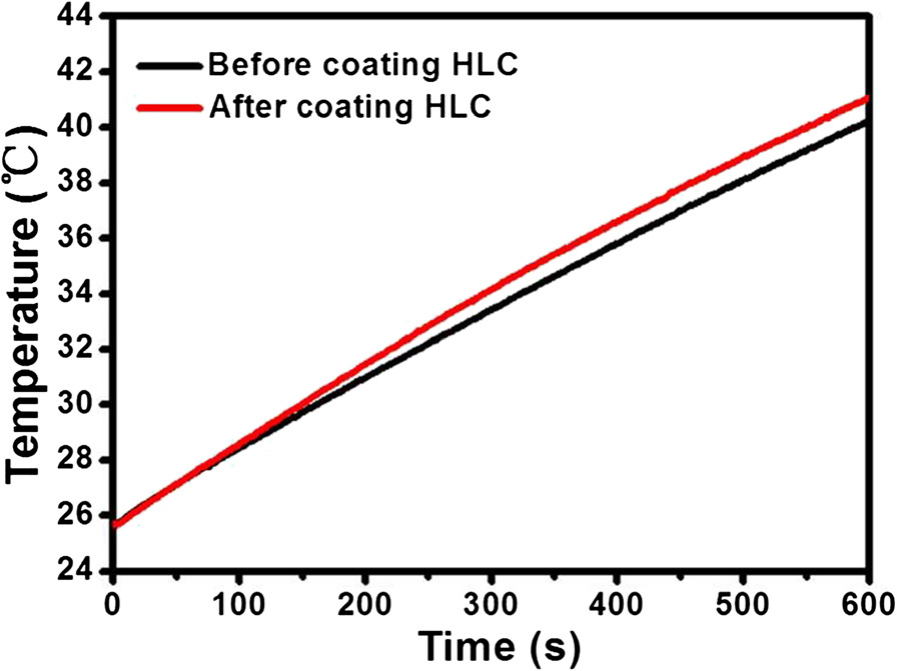
**Magnetic hyperthermia.** Time-dependent heating profile of 2 ml Fe_3_O_4_ NPs dispersion before and after coating HLC on exposure to 500 Oe alternating current field at 366 kHz frequency.

In order to assess the efficacy of HLC-coated Fe_3_O_4_ NPs as hyperthermia mediators, magnetic heating characterization was carried out using an induction heating system. As shown in Figure 6, samples before and after coating with HLC both reveal a temperature rising profile. Initially, the temperature rise profile of HLC-coated Fe_3_O_4_ NPs coincided with the sample before coating with HLC. However, after 100 s, the rate of temperature rise of HLC-coated Fe_3_O_4_ NPs was faster than that of the sample before coating with HLC. From the magnetic characterization, the reduced *M*_*s*_ of HLC-coated Fe_3_O_4_ NPs should have a slower speed of raising temperature. However, this result indicated the opposite trend. Magnetic hyperthermia is due to magnetic NPs absorbing energy from the alternating magnetic field and then, as a mediator, transform the absorbed energy to heat. After coating with HLC, the change at the NP/water interface somehow became more efficient at transferring heat, which resulted in the faster temperature rise. Moreover, the good dispersion after coating with HLC improves the Brownian contribution to heat transfer. In short, after coating with HLC, the magnetic hyperthermia performance was improved.

**Figure 7:**
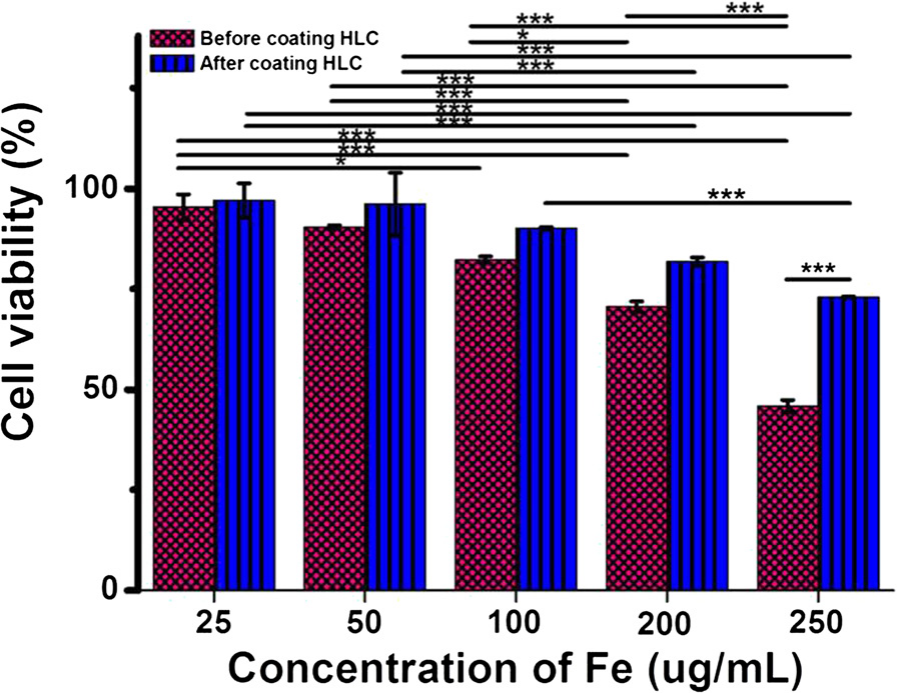
**Cell viability.** Cell viability data of Fe_3_O_4_ NPs before and after coating HLC obtained from cultured NIH3T3 cells using standard CCK-8 colorimetric assays. Error bars, SEM; *p < 0.05; **p < 0.01; ***p < 0.001.

Before being used for practical applications, it is important to evaluate the biocompatibility of HLC-coated Fe_3_O_4_ NPs. The cell viabilities were determined after a 24-h co-incubation with fibroblast NIH3T3 cells. As can be seen in Figure 7, the observed cytotoxicity increased with increasing Fe concentration. At 25 to 100 μg/mL of Fe, Fe_3_O_4_ NPs both before and after coating with HLC showed no obvious decrease in the viability of the NIH3T3 cells. Fe_3_O_4_ NPs before coating with HLC induced a cytotoxic effect in NIH3T3 cells at a concentration of 250 μg/mL Fe. However, HLC-coated Fe_3_O_4_ NPs did not induce notable cytotoxic effect between 100 to 250 μg/mL Fe. Thus, HLC has excellent biocompatibility. This means that Fe_3_O_4_ NPs after coating with HLC clearly exhibit better biocompatibility than uncoated Fe_3_O_4_ NPs, especially at high concentrations. These results suggest that HLC-coated Fe_3_O_4_ NPs are excellent candidates for further practical applications.

## Conclusions

Superparamagnetic Fe_3_O_4_ NPs were coated with biocompatible HLC to investigate their magnetic hyperthermia performance and cell toxicity. The results show that the HLC-coated Fe_3_O_4_ NPs had a faster rate of temperature rise in magnetic hyperthermia, which result from the higher heat conduction and larger Brownian contribution to heat transfer. Moreover, the biocompatibility was improved after coating with HLC. Surface functionalization of Fe_3_O_4_ NPs with biocompatible HLC gave improved stability, heating efficacy, and reduced toxicity towards normal cells, thereby enhancing the potential of magnetic hyperthermia in cancer treatment.
